# Artificial intelligence and pediatric acute kidney injury: a mini-review and white paper

**DOI:** 10.3389/fneph.2025.1548776

**Published:** 2025-02-18

**Authors:** Jieji Hu, Rupesh Raina

**Affiliations:** ^1^ Department of Internal Medicine, Northeast Ohio Medical University, Rootstown, OH, United States; ^2^ Department of Nephrology, Cleveland Clinic Akron General Medical Center, Akron, OH, United States; ^3^ Department of Nephrology, Akron Children’s Hospital, Akron, OH, United States

**Keywords:** pediatric nephrology, artificial intelligence, acute kidney injury, machine learning, hematopoietic stem cell transplantation, risk score

## Abstract

Acute kidney injury (AKI) in pediatric and neonatal populations poses significant diagnostic and management challenges, with delayed detection contributing to long-term complications such as hypertension and chronic kidney disease. Recent advancements in artificial intelligence (AI) offer new avenues for early detection, risk stratification, and personalized care. This paper explores the application of AI models, including supervised and unsupervised machine learning, in predicting AKI, improving clinical decision-making, and identifying subphenotypes that respond differently to interventions. It discusses the integration of AI with existing risk scores and biomarkers to enhance predictive accuracy and its potential to revolutionize pediatric nephrology. However, barriers such as data quality, algorithmic bias, and the need for transparent and ethical implementation are critical considerations. Future directions emphasize incorporating biomarkers, expanding external validation, and ensuring equitable access to optimize outcomes in pediatric AKI care.

## Introduction and background

Acute kidney injury (AKI) is an important diagnosis in hospitalized children as it is a frequently missed complication (1). AKI affects about 26% of hospitalized children based on the KDIGO criteria, 24% when using only the serum creatinine (SCr) KDIGO criteria, and 31% when applying both the urine output (UOP) and SCr KDIGO criteria (2). Pediatric patients who experience mild or moderate AKI are at increased risk of developing hypertension, proteinuria, and chronic kidney disease in adulthood (3, 4). KDIGO defines AKI based on SCr and UOP, an imperfect measurement (5). SCr reflects changes in glomerular filtration rate (GFR) and may take 24–36 hours to rise after a significant renal injury. These delays in the current gold standard diagnostic potentially postpone timely care.

Neonatal AKI is a distinct form of pediatric AKI, attributed to the unique physiology of neonatal kidneys and specific risk factors in this population, with NICU incidence rates ranging from 8% to 63% (6). Neonates who experience AKI face a higher risk of developing hypertension, chronic kidney disease, and proteinuria later in life (4). The Assessment of Worldwide Acute Kidney Injury Epidemiology in Neonates study further demonstrated that AKI is independently linked to longer NICU stays and higher mortality rates (7). Several conditions heighten the risk of neonatal AKI, including low birth weight, early gestational age/prematurity, hypoxic events, congenital anomalies of the kidney and urinary tract, and hypoperfusion/ischemia (8). Secondary risk factors include perinatal asphyxia, sepsis, congenital heart disease (often requiring cardiac surgery), and the increased use of nephrotoxic medications such as antimicrobials and NSAIDs (9).

The use of artificial intelligence (AI) as a method of AKI risk stratification and detection via clinical decision tools is becoming increasingly common. As technology advances, AI-driven AKI risk prediction models for pediatric and neonatal populations offer the potential to develop new risk scores and measurement tools that can identify children at risk for AKI earlier and more accurately. This white paper serves to depict current forms of AI, potential uses for AI in the diagnosis and management of pediatric AKI, and considerations for using AI.

## AI types

Machine learning is a field centered on enabling computer systems to enhance their performance through exposure to data, without relying on explicitly programmed instructions. Fundamentally, it involves the automatic detection of patterns in data, which are then used to make predictions (10). Deep learning, particularly convolutional neural networks, is an evolving field of AI that models complex data relationships and automatically detects features from large labeled datasets (11). Applied to kidney diseases, these methods have shown high accuracy and robustness across various imaging modalities. Natural language processing can analyze clinical notes to extract relevant information, enabling efficient strategies and assessments that improve diagnostic accuracy and save time (12).

Supervised learning is a type of machine learning where algorithms are trained using labeled data, meaning that the training dataset includes both features and their corresponding outcomes. The goal is to build a model that can accurately predict outcomes based on new data. Types of AI supervised learning algorithms include advanced non-linear machine learning techniques, including random forest, extreme gradient boosting, broad learning systems, elastic net final, and artificial neural networks. A systematic review in adult patients found that the broad learning system and elastic net final models had the highest pooled area under the curve (AUC) of 0.852 for predicting AKI mortality (13). A proposed clinical model based on 14-15 variables of various AI models had the highest negative predictive value, indicating its potential as a rule-out tool. These algorithms have shown potential to improve prognostic accuracy by incorporating complex variables and utilizing large datasets like Medical Information Mart for Intensive Care III and electronic health records, offering promise in reducing mortality and poor outcomes in AKI patients. Notably, random forest can be applied to a wide range of prediction issues using a relatively small number of tuning parameters, compared to other methods (14). Chiofolo et al. used a continuous random forest algorithm in adults to achieve an AUROC of 0.88 on validation, with 92% sensitivity, 68% specificity, and detection of 30% of AKI cases at least 6 hours before onset, while for AKI stages 2-3, it had 91% sensitivity, 71% specificity, and detected 53% of cases at least 6 hours before onset (15). Liu et al. reported that an XGBoost model for predicting hospital mortality in adult AKI patients achieved the highest performance with an AUROC of 0.796 (p < 0.01), an F1 score of 0.922 (p < 0.01), and an accuracy of 0.860 (16).

Unsupervised learning, another subtype of machine learning, is used to analyze and cluster data without predefined labels, meaning that there are no outcomes in the training data (17). Algorithms like k-Means, deep belief network/convolutional neural network are used. Le et al. used a convolutional neural network in adults to achieve an AUROC of 0.86 and PPV of 0.24, outperforming XGBoost and SOFA in predicting AKI 48 hours before onset, without relying on serum creatinine levels (18). Though less common in healthcare, unsupervised learning is useful for tasks that require identifying natural groupings and hidden patterns within data. Semi-supervised machine learning uses a mix of labeled and unlabeled data, training on the labeled portion while predicting and learning from the unlabeled data (19). Reinforcement learning, distinct from both supervised and unsupervised learning, involves learning through rewards, similar to psychological conditioning (20).

Most commonly, current models for AKI prediction use supervised learning; however, unsupervised models hold great potential in the future of AKI risk scores. The basics of AI algorithms and how they function, especially the difference between supervised and unsupervised learning, is particularly important to understand prior to their usage in pediatric AKI.

## Considerations for the usage of AI in pediatric nephrology

Recently, there have been many AI algorithms developed, with variations in quality and clinical effectiveness (21). The TRIPOD Initiative provides guidelines for transparent reporting in the creation and validation of such models for diagnostic or prognostic purposes (22). However, there are no widely accepted guidelines surrounding AI development and usage in the healthcare setting. AI usage should be ethical, transparent, and safe, reducing disparities in healthcare, protecting privacy, and emphasizing patient care (23).

## AI applications in pediatric AKI

AI-driven risk prediction scores for AKI have been developed across clinical areas such as critical care, surgery, and contrast-induced nephropathy to identify at-risk patients and guide clinical decisions (16, 24, 25). In pediatric nephrology, there have been a fewer number of AI algorithms developed that look solely at the pediatric population.

Improvements in AI algorithms are also being developed. For example, time series data analysis appears to be beneficial for AKI prediction models to reflect the temporal information between variables (26). This allows the clinicians to personalize treatment based on the length of time each patient stays in a hospital or intensive care unit can differ from person to person, and variations in laboratory values and vital signs that measured continuously like heart rate to laboratory values that are measured on an as-needed basis.

AI can also be used for risk stratification. For example, an AI algorithm could be applied to a validated risk scores such as the Kidney Failure Risk Equation, which estimates the 2- and 5-year risk of kidney failure using age, sex, eGFR, and urinary albumin-creatinine ratio (UACR) (27). A random forest model, applied with the simplified acute physiology score II, achieved a Brier score of 0.085 (95% CI: 0.084-0.086), an AUROC of 0.866 (95% CI: 0.862-0.870), and an accuracy of 0.728 (95% CI: 0.715-0.741) (28). By incorporating additional variables such as genetic data, lifestyle factors, and real-time monitoring of patient health metrics, the AI algorithm could provide more personalized and timely interventions. AI integrated into hospital electronic health records may also enhance clinical decision-making by analyzing patient data to provide evidence-based recommendations, predict outcomes, detect critical events early, and alert healthcare professionals to potential risks, ultimately improving care quality and reducing mortality (29).

## Identifying AKI subphenotypes

The use of AI has proven effective in specific clinical settings, such as hospital-acquired and postoperative AKI, cancer patients with AKI, traumatic injuries, and critical illness, by identifying sub-phenotypes within AKI that may respond differently to treatment based on underlying causes like ischemia, inflammation, or nephrotoxin exposure (30, 31). Moreover, unsupervised machine learning approaches could be used to stratify patients into subgroups with similar characteristics and risks.

For example, in a study that used importance matrix plots, creatinine, platelets, LDH, and diuresis were among the most influential factors across classifications (32). The consistent significance of platelets and LDH in AKI is notable, possibly due to the role of LDH in indicating liver congestion related to right ventricular dysfunction and its importance as a marker of systemic perfusion and potential hemolysis. AI has the potential to advance personalized care by incorporating individual patient lab values into tailored algorithms, enabling more precise and customized treatment strategies. Additionally, Bhatraju et al. identified two molecularly distinct AKI subphenotypes with different clinical outcomes and responses to vasopressin therapy (32).

## Implementing AI for use in pediatric nephrology

Alongside AI models for AKI prediction, numerous risk stratification tools and alerting systems have proven effective in predicting AKI. Examples include the renal angina index (RAI) score, furosemide stress test, NINJA study, and electronic health record (EHR) alerts (33–36). Deng et al. used XGBoost and logistic regression to predict adverse outcomes in 1,394 pediatric patients with AKI, achieving an AUC of 0.810 and 0.786 for 30-day outcomes, and 0.851 and 0.759 for 90-day outcomes, which included death, new kidney replacement therapy, and chronic dialysis (37). Dong et al. used a machine learning model to predict early serum creatinine-based AKI in 16,863 PICU and cardiothoracic ICU patients, achieving a median lead time of 30 hours and an AUC of 0.89 for predicting stage 2/3 AKI before detection by conventional criteria (38). The integration of these published and validated risk scores with AI (mainly machine learning) offers earlier and more precise support in routine clinical decisions. Similarly to the LOGIC score utilized for optimizing insulin dosing, advanced AI tools may aid nurses in detecting subtle changes in fluid balance and creatinine levels, thereby enhancing AKI detection earlier (39).

AI models can also be developed to predict AKI in subsets of high-risk pediatric patients, such as those undergoing hematopoietic stem cell transplantation, by identifying early patterns in risk-associated variables using neural networks (40). Risk factors for AKI in HSCT patients include patient characteristics (female sex, pre-transplant serum creatinine > 0.7 mg/dL, post-transplant weight gain > 2 kg, ICU admission), comorbidities (diabetes mellitus, hypertension, veno-occlusive disease, graft versus host disease grades 3-4, sepsis, jaundice, lung toxicity), and medication usage (etoposide-based induction, amphotericin B, aminoglycosides, calcineurin inhibitors, intravenous immunoglobulin). A proposed unsupervised AI model would utilize long short-term memory networks to create recurrent data representations as opposed to a supervised model due to the limited clinical understanding of AKI pathogenesis following hematopoietic stem cell transplantation.

The STARZ score, a validated neonatal AKI risk score, incorporates duration of stay in NICU, age at NICU entry, birth weight, the lowest temperature in the first twelve, use of caffeine, urine output in the first twelve hours, any baseline maternal/antenatal characteristics, occurrence of sepsis, and evidence of fluid overload in the first twelve hours (41). An AKI Neonatal Mortality Calculator, with a custom artificial neural network built with a Keras sequential model that incorporates the STARZ neonatal risk score, delivered impressive results with an AUC-ROC of 0.9859, accuracy of 0.9731, sensitivity of 0.9657, and specificity of 0.9805 (42).

**Figure 1:**
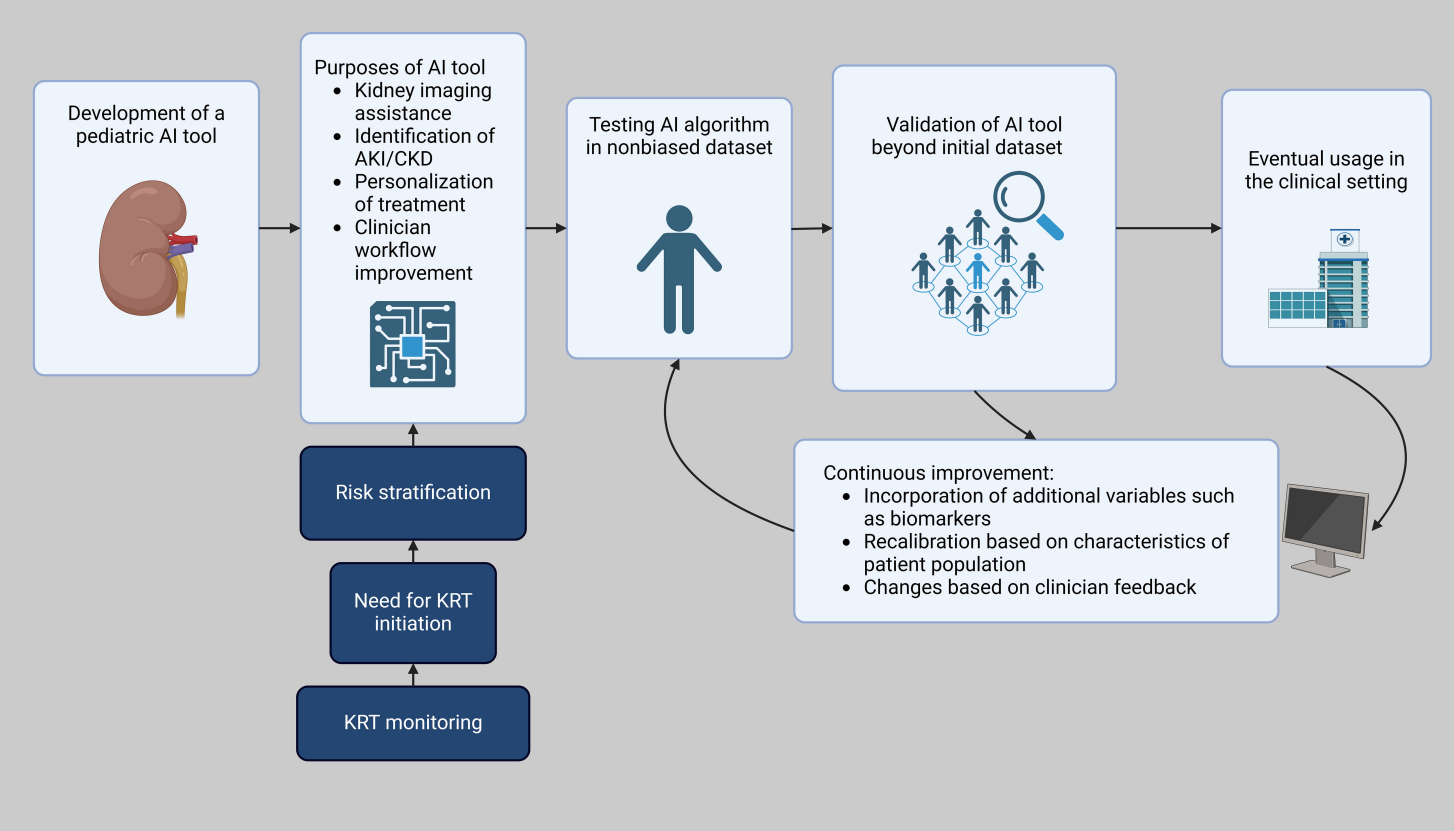
Workflow of AI integration in pediatric AKI clinical decision support. Created in BioRender. Hu, J. (2025) https://BioRender.com/b08k880.

Similarly, the Random Forest and XGBoost models achieved comparable performance metrics. AI can also be used to further analyze studies in the pediatric population. For example, in preterm neonates with AKI, mortality was independently associated with furosemide treatment in an analysis of the TINKER registry (43). AI can be used to compile a wide lens analysis to analyze the context of fluid overload, simultaneous use of other nephrotoxic agents, creatinine surveillance, other AKI mitigation strategies, specific indications, duration of use, and dosage and timing of furosemide use based on study data and assist in the formation of clinical guidelines. An example of the development and implementation of an AI pediatric AKI tool is shown in **Figure 1**. Machine learning-based models can assist clinicians in making informed decisions for critically ill patients with severe AKI by predicting adverse outcomes, including mortality and the risk of developing chronic kidney disease after discharge (44).

## Other uses of AI in pediatric AKI

**Figure 2:**
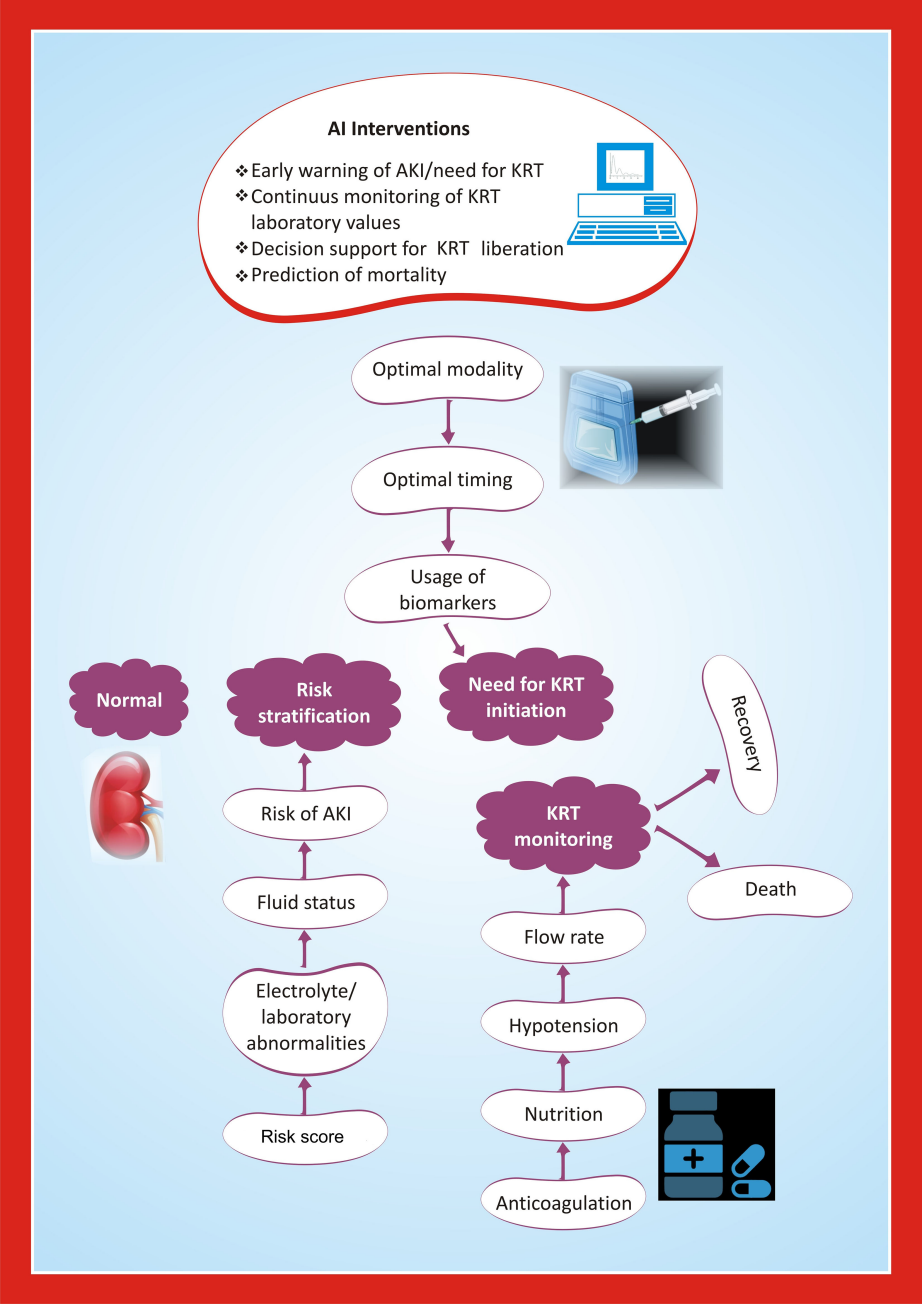
Framework for AI-enabled interventions for kidney replacement therapy in children with AKI.

AI is driving significant advancements in renal pathology and clinical care related to AKI, with applications ranging from histopathological analysis to therapeutic optimization and patient monitoring. A segmentation model with a MobileNetv3-Large backbone demonstrated high accuracy in identifying histopathological structures of acute renal tubular injury in mouse samples, achieving an overall Dice coefficient (a performance measure of segmentation algorithms) of 0.877, highlighting the potential of deep learning in advancing renal pathology evaluation (45). In patients undergoing kidney replacement therapy for AKI, AI models could integrate data from electronic health records, including fluid balance, imaging results, physiologic waveform data, and continuous kidney replacement therapy machine data, to assist clinicians in decision-making (**Figure 2**). In adults, an AI model achieved an area under the curve (AUC) of 0.70 in predicting kidney replacement therapy-free survival times for AKI patients (46). In adults, an AI model had an AUC of 0.70 in predicting kidney replacement therapy free survival times in patients with AKI (47).

AI-driven therapeutic strategies can guide drug prescriptions, reduce variability, increase the proportion of patients achieving target outcomes, and minimize errors (48). AI-driven tools in dialysis monitoring may optimize treatment parameters and improve outcomes like Kt/V, creatinine clearance, and blood pressure management without increasing patient burden (49, 50). Additionally, machine learning models can enhance clinical trials by identifying high-risk patients who may benefit from novel therapies and excluding those unlikely to respond. AI-based technologies may even be able to predict the functions of new biomarkers or drugs to treat AKI (51). These objectives are further supported by the “digital twins” approach—virtual patient representations created from multimodal data—which offers new opportunities for personalized care and may allow for identification of patients who would most benefit from a nephrologist consult (52).

## Limitations of AI

It is important for clinicians to understand the capabilities and limitations of AI, and familiarize themselves with the technology and tools (23). A major limitation of using machine learning and deep learning in healthcare is a need for high-quality, large-scale training and testing data, which is essential for reliable and reproducible predictions. Since these models learn from existing data, quality is crucial. However, feature-rich datasets are often scarce, may represent only a narrow population, and in many cases, are incomplete and inconsistent (53).

All large datasets have missing or erroneous data, which can lead to bias if not random (54). For clinical relevance, inputs and outcomes must be specific and measurable, such as defining the time frame and severity for a sepsis model. Algorithmic bias occurs when an algorithm worsens existing health disparities, making it crucial to carefully select training data and models (55). The data should reflect the target population, with performance evaluated across subgroups. Generalizability depends on the source of the data and transparency about its limitations. To truly demonstrate the generalizability of AI algorithms, external validation must be conducted using independent target populations that were not part of the original training data (56).

## Future directions

The limited available literature indicates that risk scores can be readily developed and validated using electronic health record (EHR) data, demonstrating high accuracy and generalizability (57). Modern AI applications can predict AKI before changes in serum creatinine, a century-old marker of glomerular filtration. Combining these advanced risk-assessment scores with E-alerts, care bundles, and kidney-focused interventions (such as avoiding nephrotoxins) may reduce AKI severity and its associated morbidity and mortality. Finally, incorporating biomarkers into AI models can enhance their accuracy and improve predictive capabilities, such as neutrophil gelatinase-associated lipocalin (NGAL), one of the most studied biomarkers in pediatric AKI, with urinary NGAL predicting AKI 48 hours in advance in pediatric patients after cardiac surgery (58). Other biomarkers include cystatin C, which was found to be superior to serum creatinine for estimating GFR in neonates, TIMP-2 and IGFBP, and beta-2 microglobulin (59–61). These biomarkers may improve the accuracy of AI algorithms, but require further studies. Given the limited number of pediatric AKI studies, future research should emphasize multi-center data collaboration in developing AI tools and creating guidelines to ensure AI efficacy, patient privacy, and equity.

## Conclusions

The use of AI in predicting and managing AKI in pediatric and neonatal populations represents a significant advancement in clinical care. AI models, particularly machine learning algorithms, have shown great promise in improving early detection, risk stratification, and predicting adverse outcomes in AKI patients. By leveraging both labeled and unlabeled data, AI tools can enhance the accuracy and timeliness of AKI predictions, offering clinicians crucial insights that can improve patient outcomes, reduce complications, and personalize care. However, the integration of AI into routine clinical practice requires careful consideration of its limitations, the need for transparency, and adherence to ethical guidelines to ensure patient safety and equity.

AI-driven approaches are poised to revolutionize pediatric nephrology by enhancing decision support systems and complementing traditional clinical practices. Future directions should focus on refining these models to increase their predictive power, improving their integration with existing clinical tools, and expanding their application across diverse patient populations. By addressing current limitations and ensuring alignment with ethical standards, AI has the potential to transform AKI management, ultimately improving care delivery and reducing the burden of kidney disease in children and neonates.
